# Addressing the Security Gap in IoT: Towards an IoT Cyber Range

**DOI:** 10.3390/s20185439

**Published:** 2020-09-22

**Authors:** Oliver Nock, Jonathan Starkey, Constantinos Marios Angelopoulos

**Affiliations:** Faculty of Science and Technology, Department of Computing and Informatic, Bournemouth University, Poole, Dorset BH12 5BB, UK; s4910334@bournemouth.ac.uk (O.N.); s4921588@bournemouth.ac.uk (J.S.)

**Keywords:** cyber-range, IoT, testbed, cyber-security

## Abstract

The paradigm of Internet of Things has now reached a maturity level where the pertinent research goal is the successful application of IoT technologies in systems of high technological readiness level. However, while basic aspects of IoT connectivity and networking have been well studied and adequately addressed, this has not been the case for cyber security aspects of IoT. This is nicely demonstrated by the number of IoT testbeds focusing on networking aspects and the lack of IoT testbeds focusing on security aspects. Towards addressing the existing and growing skills-shortage in IoT cyber security, we present an IoT Cyber Range (IoT-CR); an IoT testbed designed for research and training in IoT security. The IoT-CR allows the user to specify and work on customisable IoT networks, both virtual and physical, and supports the concurrent execution of multiple scenarios in a scalable way following a modular architecture. We first provide an overview of existing, state of the art IoT testbeds and cyber security related initiatives. We then present the design and architecture of the IoT Cyber Range, also detailing the corresponding RESTful APIs that help de-associate the IoT-CR tiers and obfuscate underlying complexities. The design is focused around the end-user and is based on the four design principles for Cyber Range development discussed in the introduction. Finally, we demonstrate the use of the facility via a red/blue team scenario involving a variant of man-in-the-middle attack using IoT devices. Future work includes the use of the IoT-CR by cohorts of trainees in order to evaluate the effectiveness of specific scenarios in acquiring IoT-related cyber-security knowledge and skills, as well as the IoT-CR integration with a pan-European cyber-security competence network.

## 1. Introduction

Following more than two decades of active research, the technological paradigm of Internet of Things (IoT) has now reached a high maturity level at which the pertaining question is the application of IoT technologies in systems of high technological readiness that are either close to the market or already commercialised. In this context, while the basic networking and interoperability aspects of IoT have been adequately addressed or solved, this is not the case for the cyber-security aspects.

This is nicely demonstrated by recent cyber-security incidents in IoT systems that attracted much attention. The Mirai botnet is one such example. The Mirai malware’s source code was based off the Bashlite malware and infected IoT devices, such as internet-connected security cameras, to create a botnet [1]. This botnet is interfaced by Command and Control (C&C) servers by its operators. Scanners (dedicated servers) look for vulnerable devices, and loaders (also dedicated servers) load the malware onto the vulnerable device as a payload. Malware servers host resources, such as binaries and other executables (e.g., scripts, etc.) that will be utilized by the botnet during an attack. The Mirai botnet is an example of an exploitation of the LAN Mistrust problem domain. The botnet was used in October 2016 to conduct a Distributed Denial of Service (DDoS) attack against people’s routers across the world and caused many of the most popular websites at the time to be rendered unavailable [2]. This attack served as a proof of concept that IoT devices could be utilized in wide-scale networking attacks and derivatives of the Mirai malware have been found since. Additionally, in 2016, researchers were able to remotely access a Tesla Model S from a distance of 12 miles away [3]. They were able to interfere with functionalities ranging from car door locks, to the car’s breaks and dashboard computer system due to these and other functionalities being electronically controlled, and stemmed from (according to Tesla) the car’s web browser being used whilst connected to a malicious Wi-Fi hotspot. This is another example of exploitation of the LAN Mistrust problem domain.

The aforementioned examples highlight the significant gap that currently exists in terms of cyber-security competencies in IoT [4]. If the discrepancy between this skills-shortage and the required security expertise becomes too large, this may result in a decrease of security as-a-whole in IoT environments, with a subsequent increase in cybercrime. The exponential growth of IoT has led to the commissioning of millions of new devices, which are collecting and transmitting information but with many vendors not conforming to security best practises. This means there are millions of potentially vulnerable devices which could be exploited, thus revealing new vectors of attack. There is a dire need for dedicated infrastructure to be used for evaluating and training cyber-security competencies in IoT.

A respected method of training an individual and increasing their competency is via practising scenarios on a cyber range. This allows for trainees to experiment and hone their abilities to deal with situations that make occur in a safe, and isolated manner. Cyber ranges also provide suitable infrastructure as a test-bed allowing experimentation. It is noted that there are many cyber ranges which are useful for the study of cyber security, and that there are many test-beds which allow for the testing of IoT devices and networks. Paradoxically however, there is very little literature regarding IoT cyber ranges and security.

Our contribution. We address the existing and ever increasing problem of skills-shortage and lack of research infrastructure focused on cyber-security in IoT. We present an IoT Cyber Range; i.e., an IoT test-bed that is designed to support research and training on cyber security aspects of IoT systems and networks. The design is focused around the end-user and is based on the four design principles for Cyber Range development defined by Schwab and Kline [5]. The architecture is modular, consisting of a front-end and a back-end that are loosely coupled via a RESTful API. This obfuscates the underlying complexity of the back-end from the end-user, while at the same time isolating the front-end from future extensions in the supported IoT technologies and system architectures at the back-end. The architecture is scalable, allowing for multiple users and sessions running concurrently. This is achieved by leveraging upon Cooja-a state of the art emulator of IoT networks-that is run in headless mode. Each user is able to specify their scenario (network topology, configuration and IoT application developed in Contiki-NG) and submit it for execution; then, the system provides them with log files detailing the emulation. We demonstrate the use of the facility via a red/blue team scenario involving a variant of man-in-the-middle attack using IoT devices.

The rest of this paper is articulated as follows. Section 2 reviews the existing related literature including existing IoT experimental facilities, federations of such facilities and cyber ranges. Section 3 explores the IoT Cyber Range architecture and available official security guidance on development of cyber ranges. Section 4 describes the technical implementation of the front-end engine and user interface. Section 5 describes the technical implementation of the IoT Cyber Range engine in the back-end. Section 6 discusses scenarios for cyber security training and demonstrates a proof-of-concept. Section 7 concludes this work by summarising our contribution and providing insights of our future work.

## 2. Related Work

### 2.1. Cyber Ranges

There are varying definitions of what constitutes a cyber range. Yamin et al. in [6] define a cyber range as an environment providing testbeds for research and conducting training through programs. Alternatively, Kavallieratos et al. in [7] describe a cyber range as an interactive, simulated representations of an organization’s local technical infrastructure connected to a simulated Internet. They provide an isolated, and safe environment to legally practise security training without the risk of consequence.

According to Ficco and Palmieri [8], cyber ranges can consist of physical infrastructure, be completely virtualised, or a hybrid between the two. The suitability of each option depends on the wider context on which the cyber range is being built. Yamin et al. [6] also argue that there are 6 aspects that are needed for a cyber range to be considered fully functioning and effective. These are monitoring capabilities, learning, management, teaming, the environment, and scenarios. Monitoring capabilities allow for the observation of participants for effective learning, and that the cyber range is performing to an acceptable standard. This involves designated observers using methods, tools, and layers of which monitoring is being performed. Learning involves the scoring of users which means one can determine whether the cyber range is an effective learning tool. Management involves the “assignment of roles and duties to individuals and teams”, which includes role management, resource management, and range management. The teaming aspect refers to the groups and individuals who are involved in the creation and participation of cyber range scenarios. This includes the red team (attacking), blue team (defending), white team (designing of scenario), green team (monitoring and maintenance of scenario infrastructure), amongst others. Environments contain the services which are used to support scenarios. These can be virtualised, physical, or a hybrid of the two [8]. Scenarios define the execution environment, context, and additional background information and stories behind exercises which are used to test individuals. These combined aspects define the characteristics of a contemporary cyber range.

### 2.2. IoT Testbeds and Cyber-Ranges

In [9] authors survey multiple IoT testbeds and experimenting facilities to identify mutual requirements. Authors found that most testbeds need to scale appropriately to facilitate their requirements. As IoT becomes truly global, a global-sized infrastructure will be needed to allow for full scale experimentation. This could be offered through federation or virtualisation of nodes. The heterogeneity of IoT devices in various contexts needs to be replicated in experiment infrastructure to provide a realistic and credible scenario. There is also a need for repeatability of experiments to validate results. This means that experimentation parameters need to be recorded in a way that can be shared with others. Concurrency of multiple users and experiments is necessary to make a testbed economically viable and allow for further investment and research. Increased robustness of experimental infrastructure allows for moving of cyber ranges towards a more credible and realistic experimental environment.

In [10], authors describe OpenTestBed; an open-source testbed, with comparatively cheap setup costs, replicable with its open-source and off-the-shelf nature of its components. It is simple in its nature by generically logging information through serial connections to best facilitate a variety of tests. It’s physical architecture consists of a Raspberry Pi, 4 OpenMote B motes, a screen for output, and a QR code. The Raspberry Pi simply acts as a central server running a single python program. These components are grouped together to form an “OtBox”. It is not a federated testbed but multiple “OtBoxes” can be grouped together allowing for data aggregation via an MQTT broker. There is also support for integrating OpenTestBed into OpenWSN allowing for application of the serial data by external tools. The focused aspect of this testbed is to serve as a proof-of-concept that dedicated testbeds and cyber ranges can be developed cheaply compared to traditional institutionally dedicated testbeds. However, the user interface is limited and generated data likely homogeneously.

The authors in [11] present the KYPO4INDUSTRY cyber range which was designed to address the cyber security skills gap within industrial control systems (ICS). The training facility is ideal for beginner and intermediate computer science students in a simulated industrial environment. The testbed consists of a physical setup of ICS hardware nodes, such as PLCs, memory, and peripheral devices, interconnected by an isolated network. Authors in [11] further propose a course alongside the testbed which is designed to “provide an awareness of threats within the ICS domain with practical experience”. This course is the equivalent of 13 weeks of dedicated study and involves content ranging from motivation, real attacks, and legal issues, to threat modelling, creating, and deploying an ICS Capture-the-Flag (CTF) game. This course serves as an evaluation of the testbed’s effectiveness in teaching, but the paper does not state the results from course participants, denying any potential scrutiny.

### 2.3. Federated Testbeds and Cyber-Ranges

GENI (Global Environment for Networking Innovations) is a scalable infrastructure and provides services such as a virtual laboratory for conducting large-scale network experimentation via a federated architecture [12]. It is open source and is maintained by a community of stakeholders [13]. Some research argues that the concepts of “sliceability” (virtualisation and simultaneous sharing of resources while maintaining a degree of isolation for separate experiments), and deep programmability (the ability to influence low-level behaviours and interactions for the purpose of modifying to an experiments context) expands the potential for experimental networks [14]. Its focus on large-scale, federated networking solutions is unique amongst other testbeds, existing on an international domain. However, these solutions don’t relate to any dedicated IoT paradigms. It is argued that due to GENI’s varying range of experiment styles, durations, and sizes there is no single experiment interface. To solve this, GENI allows for interfacing through multiple APIs. The research continues to argue that forgoing the benefits of a single user interface (standardization, concentrated support, etc.), is a purposeful strategy which encourages the development of interoperating developer tools [14]. Though it should be stated that while initial results seem positive, it is too early to draw any conclusions.

FIESTA-IoT (Federated Interoperability Semantic IoT Testbeds and Applications) provides a federated infrastructure to allow for the experimentation of heterogeneous IoT technologies through an “experiment-as-a-service” solution [15]. The platform provides access to 10 testbeds across multiple countries with the capability of further scalability. FIESTA-IoT is designed to solve issues pertaining to isolated data from testbeds in different industry sectors. [16] describes how the platform allows for the translation of data to a common FIESTA-IoT ontology via a common API. Effectively the platform works as a proxy for its federated testbeds to proving a common standard for access of data allowing for the semantic interoperability of these testbeds, access of corresponding data streams and plug-ins and discovering of resources. The focus of the testbed is on the scalability and interoperability of IoT devices on a large scale and aims to confirm or deny the functionality of these devices and infrastructures, with the architecture following a modular approach. A limitation to this federated cyber range is that there are no dedicated security testbeds. This is significant regarding the large security hole that IoT devices and systems currently entail. Within this testbed, there is a lack of security related services. Experiments are deployed utilizing the system’s testbed services. There is no mention as to whether these are scheduled or queued.

The FIRE (Future Internet Research and Experimentation) and its successor (FIRE+) provides a federated infrastructure where multiple testbeds and platforms allow for the connection between research and large-scale experimentation [16]. This testbed allows for research and experimentation into the field known as Future Internet where one studies the internet’s prospects and emerging related technologies. FIRE(+) focuses on the management of diverse resources and facilitates the experimental life-cycle in areas such as sensor networks, IoT, 5G, SDN (Software Defined Networking), and Cloud Computing etc. As part of this federated solution, there is no dedicated IoT testbed for security-specific training and experimentation. The testbed’s focus is similar to GENI, but based in Europe. During the design process, careful attention was given in order to align to the GENI testbed in the US, allowing for interoperability between the two testbeds. The 4 key areas of discussion were identified as “resource discovery, reservation, and provision”, “monitoring and measurement”, “experiment control”, and “SLA management and reputation services” [17]. There is no mention of security-focused or security-specific evaluation of technologies.

There are multiple, entirely virtual cyber ranges that allow for the practical training of cyber security through specific scenarios. The emergence of the cloud paradigm has allowed for the industrial scale access to virtual machines across the internet allowing for users to train against specific scenarios on-demand. The on-demand nature and scale of these solutions allows for a variation of scenarios covering differing topics from IoT best practices for security, to secure coding practices, configuring cloud solutions like AWS securely, differing aspects of web security, penetration testing, malware analysis, open-source intelligence, etc. The architecture consists of a cloud back-end, with a central portal for access. ImmersiveLabs [18] offers a dedicated virtual cyber range solution to educational institutions with a competitive aspect in terms of intra- and inter-university leader boards. HackTheBox [19] encourages users to hack its front page in order to gain an invite code to sign-up, with similar competitive leader boards. TryHackMe [20] allows for multiple users to form teams in formal cyber security competitions (such as the HackBack CTF). HackTheBox and TryHackMe both require access to a VPN via OpenVPN clients in order to safely run the scenarios on an isolated network. Neither of these virtual solutions offer a dedicated IoT-related security testbed as part of a federated solution.

There also exist four flagship projects, funded by the European Union as part of the Horizon 2020 program [21]. These projects are designed to work simultaneously and to complement each other with closely related objectives and goals. The ECHO project [22] is described as a system of federated cyber ranges designed to increase the competency of cyber security within the European Union. The CONCORDIA project is a cybersecurity competence network providing an ecosystem to lead research, technology, and industrial and public competencies [23]. Similar to Concordia [23], SPARTA is another cyber security competence network aimed to coordinate research, innovation, and training within the European Union [24]. CyberSec4Europe is a research project focused on the implementation of potential government structures in order to create a European Cybersecurity Competence Network with an emphasis for best practise examples [25].

### 2.4. Existing Guidelines on Cyber Range Development

There is little official guidance from the NCSC (National Cyber Security Centre) or ENISA (European Union Agency for Cybersecurity) regarding the design and development of cyber ranges. ENISA have stated that cyber ranges for the development of competencies is an agenda item [26], so there is the possibility that guidelines could be developed in the future.

In [5], authors specify four principles for building cyber ranges which are focused on cyber security. These principles were developed with years of experience in developing cyber security testbeds. They are defined as follows:

1. Provide tools and capabilities that reduce the cognitive burden on experimenters wherever possible.
2. Allow experimenters to encode their goals and constraints and leverage this information to help guide experiment construction.
3. Provide flexibility in design. A good architecture evolves with both its users and technology, and newly developed capabilities.
4. Provide multifaceted guidance to help experimenter produce high-quality experiments.

Principle 1 states that using tools to allow the transfer of knowledge between people reduces the cognitive burden on experimenters allowing them to concentrate on their tasks at hand. Principle 2 states that goals and constraints should be included in the experiments’ design requirements to help focus the construction to meet these requirements. Principle 3 states that architectures evolve with their users, and newly evolved capabilities should be designed with leeway in mind to provide flexibility to meet these changing requirements. Principle 4 states that guidance should be available from all angles to help stream-line design and implementation of good-quality experiments and scenarios. This includes automated or human-driven guidance. These principles offer some level of detail but are ambiguous in their application with no guidance from the authors on how to apply these rules in practise. Having this guidance available would allow for novice experimenters transition into well-experienced experimenters with the necessary skills and knowledge to create high-quality experiments.

Additionally, NIST (National Institute of Standards and Technology) suggests in [27] that there are specific properties of a cyber range that should exist in order for the cyber range to be considered of a high standard. These include technical components (learning management system, target infrastructure, virtualisation layer, etc.), accessibility and usability (i.e., a cyber range should be both useable and accessible to its target audience), scalability, etc. Scalability allows for potential growth to enable new scenarios and provide additional flexibility and agility to rising threats and new scenarios.

## 3. IoT Cyber Range Architecture

This section elaborates on the high-level architecture of the system. Section 3.1 covers existing official security guidance, or lack thereof, for developing cyber ranges and testbeds. Section 3.2 explores sequence diagrams to help visualise user interactions with the system for example tasks. Section 3.2 details a high level overview of the architecture of the system including both the front-end and the back-end. The selected approach for the development of this artefact is IBM’s RADM approach. IBM [28] recommend using RESTful API Design Model (RADM) for RESTful API model-driven development which allows for one to describe an API, its contents, and the technical structure of the model. The models are UML-based, and support the four stages of RESTful API development life-cycle. In an iterative fashion, one elicits API requirements, builds the API models, generates API specification and pseudo-code, and then implements and manages the API. This approach allows for the quick development of meaningful functionality and provides value to projects in a fast turnaround environment. Its individual steps of the iterative cycle are simple, and it allows for an adaptive development process to potentially changing requirements. The models created as a result of the RADM approach will be shown in this section via sequence diagrams to show use-case examples of API interactions.

### 3.1. User Interface

To enable the elicitation of requirements, this section explores actions which need to be conducted in order for the web interface to function. Below sequence diagrams are presented which explore the interaction between the user and the system, and the sequence of steps and interactions when performing a key task. From these diagrams one can explore required functionalities which allows for functional requirements, and supportive requirements.

#### 3.1.1. Resource Provisioning

**Figure 1:**
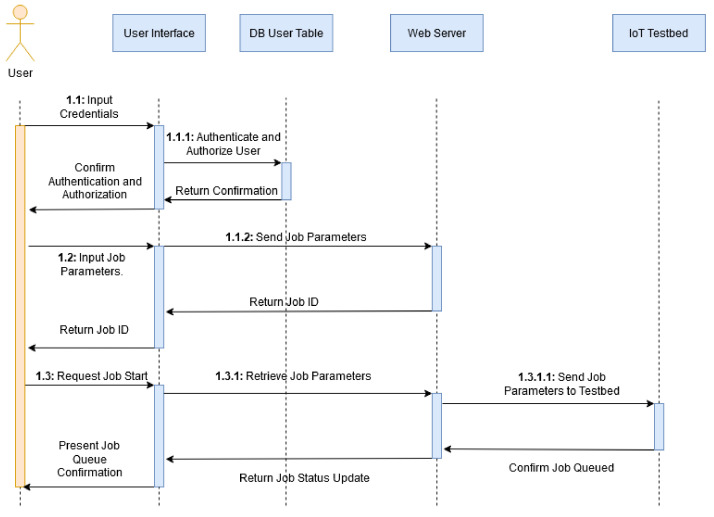
Job Queueing Sequence Diagram.

The below sequence diagram depicted in Figure 1 shows the steps taken and interaction between the user and different layers of the system for queuing a job onto a resource node. This involves authentication (where the user credentials are verified) and authorization (where use privileges are verified) via interaction with the user interface and database, and the selecting of jobs and forwarding to the IoT Cyber Range. The job status is then updated and returned to user as confirmation of a successful job run. Indexing shows when a sub-action is part of an action.

#### 3.1.2. Retrieving Experiment Logs

**Figure 2:**
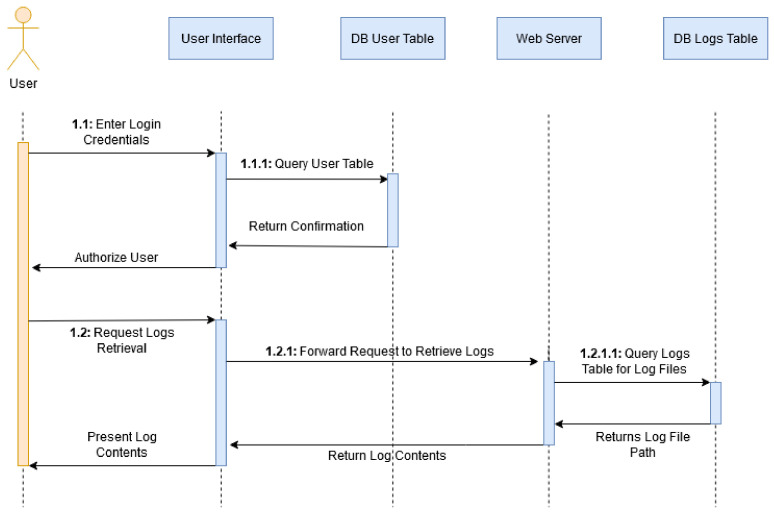
Log Retrieval Sequence Diagram.

The sequence diagram depicted in Figure 2 describes the interactions between the user and the system when requesting logs pertaining to a job. The user enters their login credentials which are queried against the user table in the database to return a confirmation that the user is authorized to proceed. The user can then request the retrieval of logs which is forwarded from the interface to the Logs table with a search parameter specifying the log ID. The logs are displayed to the user. Indexing shows when a sub-action is part of an action.

#### 3.1.3. Inquiring Resource Availability

**Figure 3:**
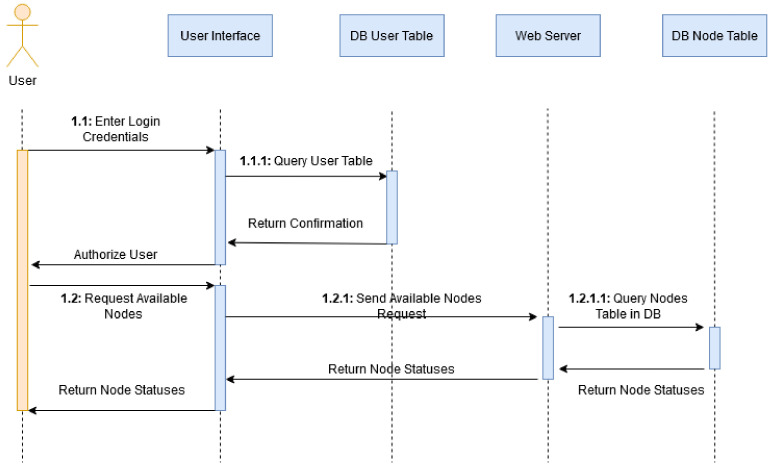
Retrieving Available Nodes Sequence Diagram.

The below sequence diagram (Figure 3) describes the actions and interactions between the user and the system when requesting node statuses and their availability. This includes the authentication of a user and the forwarding of the user’s subsequent request to the testbed. The user enters their login credentials which are then queried against the user table in the database. The confirmation is returned, and the user is authorized.

The user can then request the node states. The interface queries the back-end, then queries the nodes table returning the number of available nodes both physical and virtual. Indexing shows when a sub-action is part of an action.

### 3.2. Architecture Block Diagram

**Figure 4:**
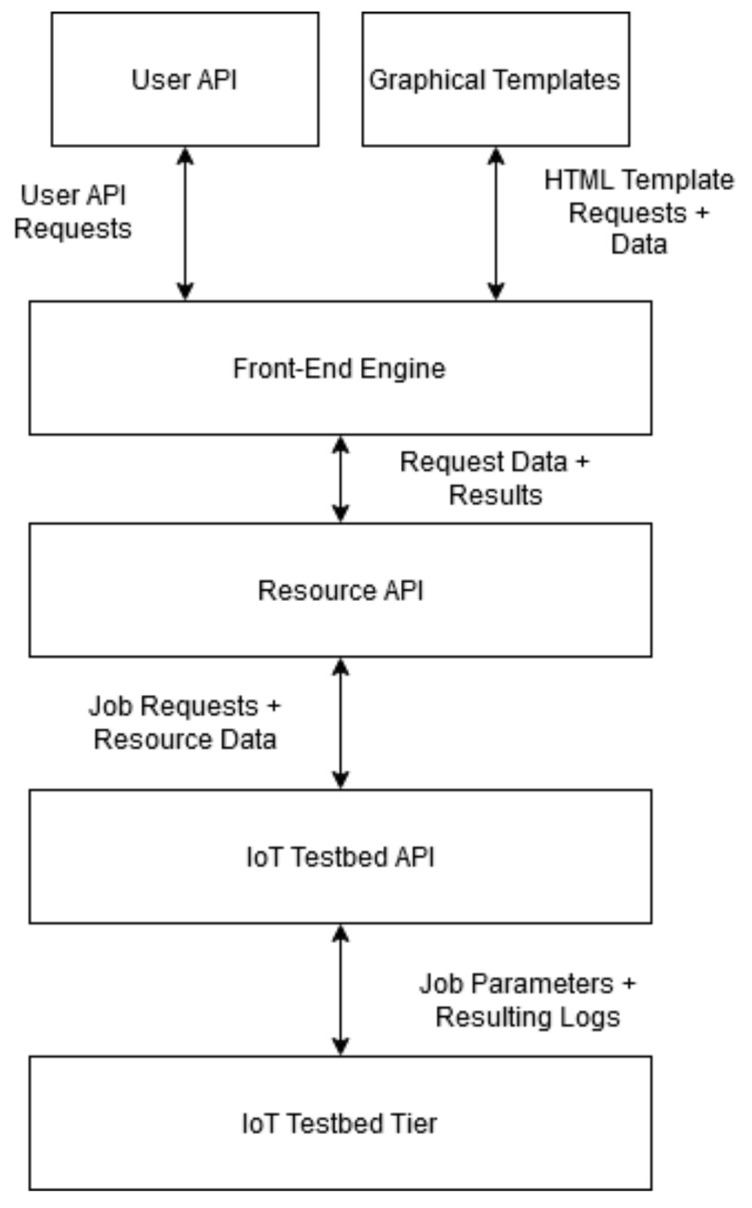
System UML Block Diagram.

**Figure 5:**
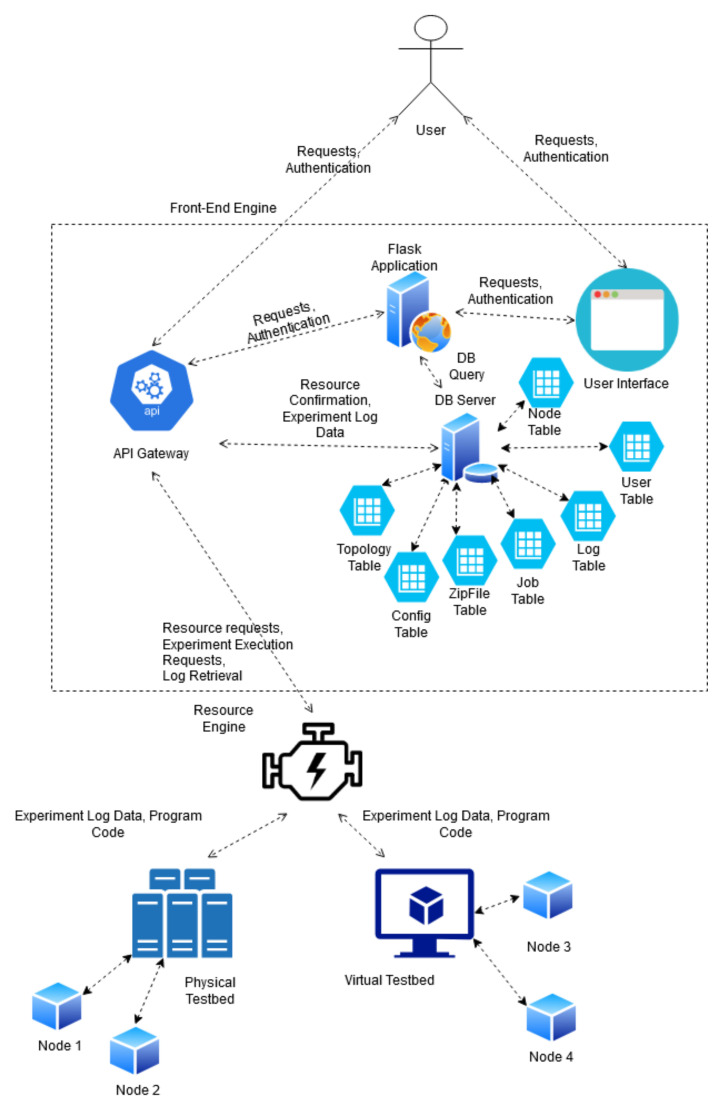
System Architecture of the IoT Cyber Range.

The below diagrams (Figure 4 and Figure 5) depict the high-level interaction between different modules of the system. At the north end exists the user interface tier which consists of a user API and graphical templates allowing for interaction between the testbed and its users. These two interfaces are maintained by the front-end engine which handles customer requests, and authentication. The Resource API exists at the south end of the front-end engine in order to allow for communication from the IoT testbed tier when updating node statuses or sending back logs. On the opposite end of this communication line exists the IoT testbed API which allows for the receiving of job parameters from the Resource API. This API provides an additional layer of abstraction from the physical and virtual testbed resources in the IoT testbed tier. At the lower tier, the facility comprises both physical and virtual IoT resources. In particular, the physical component consists of twenty RE-MOTE IoT devices provided by Zolertia [29]. The devices form a wireless peer-to-peer mesh network over IEEE802.15.4 and are connected to the IoT-CR server over a USB-tree cabled topology for management purposes. The virtual component is powered by Cooja; a network emulator using the Contiki-NG embedded OS.

## 4. Front-End User Interface

This section explores the low-level design, implementation, and testing of the system application’s front-end. This includes tools and libraries used in development, the full layout of the system architecture, Object Orientated Programming (OOP) class diagrams, the schema of the API structure, web interface designs, highlighted code snippets, and testing including unit testing and user testing.

### 4.1. Selected Tools for Development

The Web Interface and API to the testbed is written in Python 3.7 [30]. The IoT testbed tier is being written in Python. Using Python to construct the Web Interface allows for easier integration of services between the front-end engine and the IoT testbed tier. Further to this, the Python syntax is easy to read and quicker to type allowing for a faster development time. Speed of execution is not a priority for this project so this is not a bottleneck for implementation. Python facilitates importing of other codebases. In particular a codebase called Flask [31] which is a lightweight web framework allowing for the rendering of API structures, HTML templates, and back-end functionality. It also supports interfacing to databases via the flask-sqlalchemy dependency. HTML5 [32] templates and CSS3 [33] were used for the creation of graphical interfaces with bootstrap (Otto et al., 2020) also being used to provide an adaptive interface layout to different resolutions. This would allow a user to view the graphical interface on a mobile device if required. Further to this, Curl [34] is used to test API calls. Git [35] is used to allow for version control of the software code, as well as providing a remote backup for the project code as per risk analysis. SQLite3 [36] is used as a lightweight means of persistent data storage. SQLite3 does not require a Database Management System (DBMS) to function as it works as a stand-alone file. This provides efficiency for system resources in terms of storage space and resource memory.

### 4.2. Database Design

**Figure 6:**
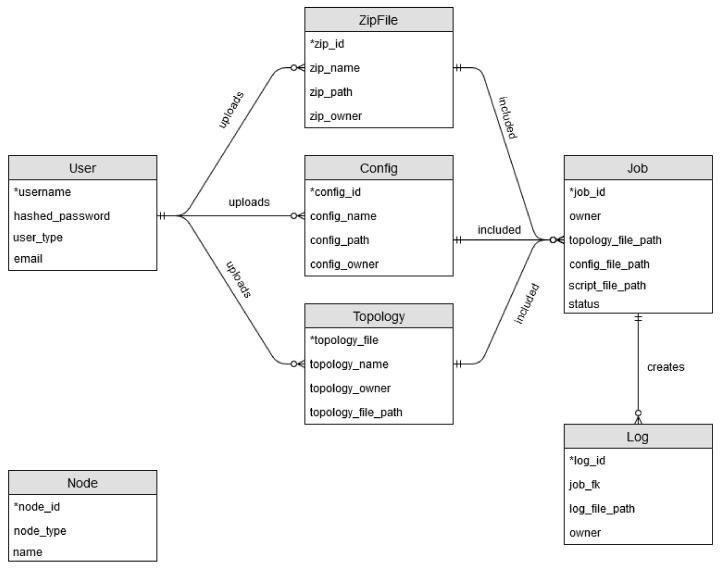
Log Retrieval Sequence Diagram.

The ERD depicted in Figure 6 consists of seven tables: User, ZipFile, Config, Topology, Job, Log, Node. The User table holds user information such as username, the user type, etc., and is utilized during authentication and authorization. The ZipFile table handles information pertaining to ZipFile details. Config pertains to configuration information, and Topology pertains to topology information. These 3 tables link to the Job table which combines the 3 files. From a Job, one or more logs can be created. The Node table is unrelated to the other items and pertains to nodes which are currently available to the users.

### 4.3. Api Structure

The base URL of the API will be “/api/v1/” to allow for an API version control framework. It uses a JSON schema. The API URL structure is as follows:

Authentication-‘/api/v1/auth’ The user must be able to authenticate. This endpoint takes a POST request with JSON data requiring a username, and password field. A successful POST request will allow for the creation of a JSON Web Token. A ‘username’ and ‘password’ key will be required as data.

Sign Up-‘/api/v1/signup’ This endpoint allows a user to create an account by sending a post request containing the following keys: ‘username’, a potential ‘password’, and an ‘email’ address. This endpoint returns a message stating a successful sign-up, or a failed sign-up if the username is already taken. A user by default determined as a “customer” type. Currently in order for a user to be deemed an admin, or a system user type, they must contact the admins of the testbed.

Available Nodes-‘/api/v1/nodes’ A customer may send a GET request to this endpoint in order to retrieve the number of physical and virtual nodes available. This use case returns a message stating the number of each. If the user is a system user then they have the ability to send a POST request to add a node. This needs to include a name of the new node, and its ‘node_type’, either “virtual” or “physical”. A DELETE request with a ‘node_id’ key allows for the deletion of nodes rendering them no longer available.

Topologies-‘/api/v1/topologies’ The user must be able to run an experiment, and the topologies file is part of this. The topologies endpoint allows a user to view their uploaded topology files showing their filename and their associated ID. Sending a POST request allows for the uploading of another topology file with a ‘file’ key. A POST request would return a message stating the file has been uploaded with an associated ID, or that the file failed to upload as it already exists.

Configs-‘/api/v1/configs’ The ‘configs’ endpoint allows a user to view their uploaded configuration files showing their filename and their associated ID. Sending a POST request allows for the uploading of another configuration file with a ‘file’ key. A POST request would return a message stating the file has been uploaded with an associated ID, or that the file failed to upload.

Scripts-‘/api/v1/scripts’ The user should be able to upload scripts to facilitate an experiment. Scripts need to be uploaded as .zip files. The scripts endpoint allows a user to view their uploaded zip files showing their filename and their associated ID. Sending a POST request allows for the uploading of another zip file with a ‘file’ key. A POST request would return a message stating the file has been uploaded with an associated ID, or that the file failed to upload.

Jobs-‘/api/v1/jobs’ The jobs endpoint allows for a user to view their created jobs and their ID’s by sending a GET request. Sending a POST request with an uploaded ‘topology_id’, an uploaded ‘config_id’, and an uploaded ‘zip_id’ will result in the creation of a new job and an ID will be returned. A GET request to see a specific job file can be done by sending the request to ‘/api/v1/jobs/<job_id>’ where <job_id> is the ID of the job. A job can be run with a GET request sent to ‘/api/v1/job/<job_id>/run’ where <job_id> is the ID of the job to be run.

Logs-‘/api/v1/jobs/<job_id>/logs’ One can see log IDs associated with a job by viewing the endpoint above with a GET request. A system user can send a POST request to add an additional log which includes the logfile (‘file’) as a key and the job_id its associated with. The file needs to be a zip in case multiple logs are returned. A user can see log contents be sending a Get request to ‘/api/v1/jobs/<job_id>/’. A successful POST request will allow for an email to be sent to the user who requested the job to be run, letting them know that logs are ready to be observed.

Help-‘/api/v1/help’ A user can send a GET request to view helpful information from this endpoint. Further help is also available by some additional endpoints detailed within the help information.

### 4.4. API Python Wrapper

**Figure 7:**
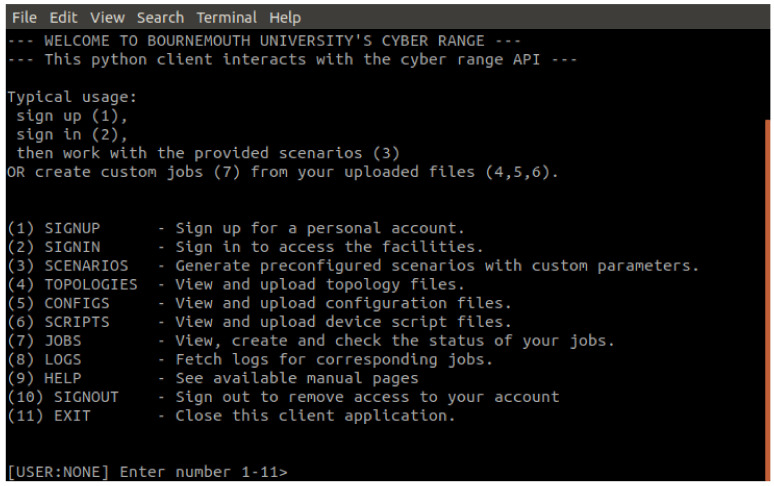
The home screen of the wrapper.

To aid users in consuming the API, which would otherwise require lengthy curl requests, a lightweight python3 client wrapper is implemented Figure 7. This wrapper allows the user to sign up and login to the Cyber Range by operating on the API endpoints presented in Section 4.3 through a menu driven interface. The wrapper holds the JTW token associated with the logged in account, allowing users to query their files, jobs and fetch log files. Below are screenshots of the wrapper in use, creating a job from a scenario, running the job and collecting the log files.

## 5. IoT-CR Back-End Operation

In order to satisfy the various needs of IoT researchers, the cyber range is virtualised to offer a range of devices that would not otherwise be accessible in physical form. The presented Cyber Range is powered by Contiki-NG-probably the most commonly used embedded OS for IoT networked systems- and Cooja; a network simulator for Contiki-NG written in Java and designed specifically for Wireless Sensor Networks. Cooja is a tool provided within the Contiki-NG Operating System, which itself focusses on “dependable (secure and reliable) low-power communication and standard protocols for IoT, such as IPv6/6LoWPAN, 6TiSCH, RPL, and CoAP” [37]. Cooja provides a detailed emulation of the entire network stack (from the link layer to the application layer) using as input the same Contiki-NG source code that would be deployed in actual physical IoT systems. This enables the researcher to focus either on individual devices or on the networking aspects of a system. Furthermore, it provides great agility to the IoT-CR as scenarios developed to be run virtually can be easily ported to physical IoT systems.

Cooja is able to operate using the scripts and libraries available within Contiki-NG, providing a graphical framework for users to assign custom scripts to virtualised devices, control these devices within a topology map and test networking scenarios with the provision of tools such as viewing real-time device standard output or adding breakpoints within the simulation for key events.

**Figure 8:**
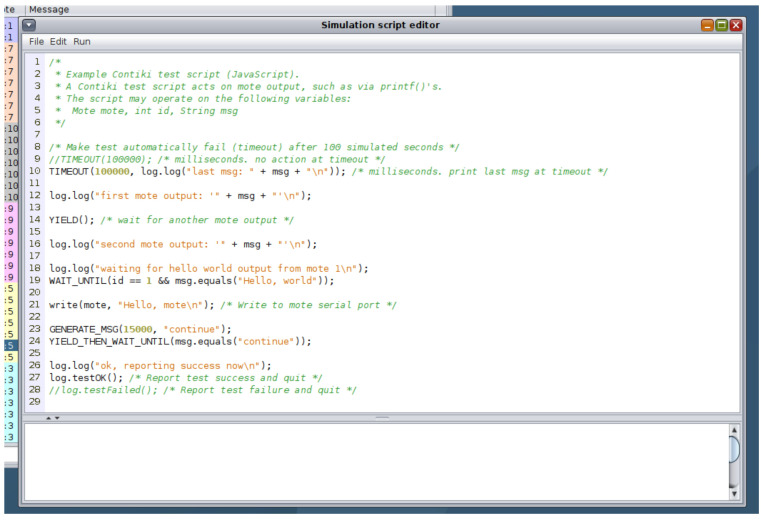
Simulation Script Editor with example JavaScript Code.

Cooja also has the ability to run in headless mode (i.e., with no graphical user interface) thanks to an inbuilt debug mode. Cooja allows simulations to be saved in ‘.CSC’ format, saving all the parameters for the simulation in XML. Whilst this can have the typical usage of saving and reloading a simulation, the CSC files can also be passed to Cooja via the command line to force a non-graphical Cooja process. Furthermore the ‘Simulation Script Editor’ tool provided within Cooja allows for users to have scripted control over the simulation via JavaScript code-seen in Figure 8.

The Simulation Script Editor allows the user to save the simulation CSC file amended with the JavaScript code as another XML element, allowing the simulation to run automated, without the need for manual intervention. Users can create their own code or use a number of preconfigured scripts. This allows for a logical detachment from Cooja, steering the Cyber Range away from simply providing Cooja as an Application-as-a-Service to utilising Cooja’s simulation abilities to provide users with a Cyber Range experience similar to other IoT testbeds. As the Simulation Script Editor is fully integrated within Cooja, and therefore Contiki-NG, all system defined events (YIELD, PROCESS, YIELD_THEN_WAIT_EVENT_UNTIL, PROCESS_WAIT_EVENT_UNTIL, etc.) [38] can be detected and triggered within the JavaScript code.

Given the simulated nature, rather than emulated, the system can run on servers capable of handling multiple jobs, as not much computing power is required to emulate IoT devices that are typically resource constrained. Furthermore, virtual systems offer scalability beyond physical systems due to the hardware independent properties, but can restrict the experience in such cases as interacting with physical buttons or sensors. Cooja uses the hosting Operating System to maintain the resource allocation required for simulations, so future development allows us to operate on hosted server-grade hardware for improved performance with costs dictated by system usage rather then maintenance of physical devices.

The automated handling of Cooja, dictating the control and running of jobs, is written in Python 3. This script polls the database periodically for newly submitted jobs. When new jobs are run in Cooja the logs, standard output and standard error are captured by default, as well as any logs generated by the user within their simulation script code (the JavaScript code). These logs are then exposed over the API for users to consume.

## 6. Demonstration of a Cyber-Range Scenario

**Figure 9:**
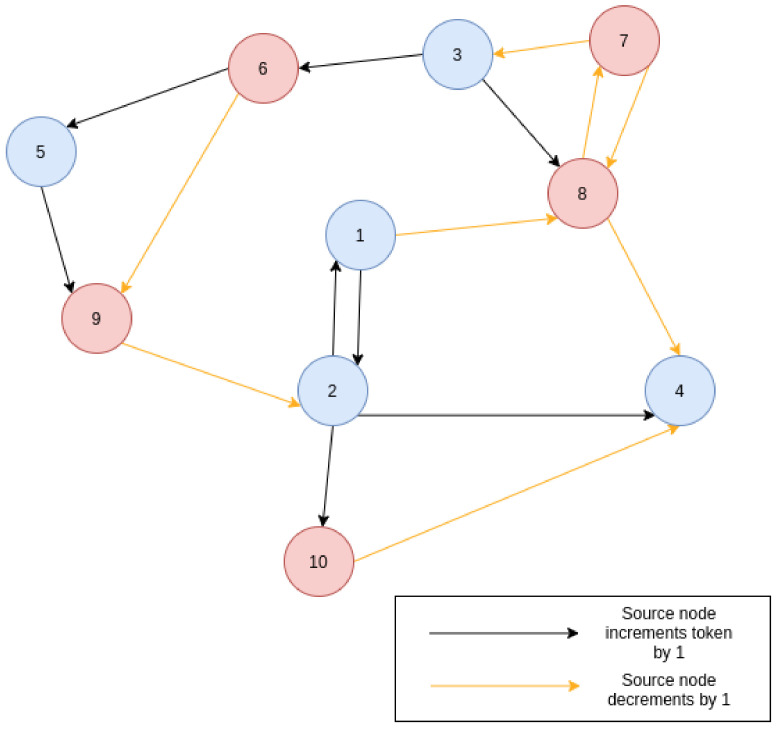
Ten-node network depicting the token modification and exchange.

In order to enable initial user comprehension and to enable quicker job development, the system can provide scenarios that offer the complete Contiki-NG project build folder and Cooja simulation (CSC) file required for any job. Generating these scenarios, users will be able to understand the format of the simulation code in use, providing the opportunity to change the parameters of a given scenario such as: wireless protocols, quantity of nodes, density of network, percentage of nodes running particular code (accounting for sink nodes or different team sizes). In Section 6.1, the example user generates the code for the scenario “Pass the token”. This scenario demonstrates typical blue team/red team cyber security events. The scenario plays out as two opposing teams trying to share a token value; whilst the blue team attempts to increment and share the token between themselves, the opposing red team attempts to intercept and decrement the token before passing it onto the blue team, resembling a simplistic man-in-the-middle attack. Blue team tries to increment the value resembling a defence response. No node may send more than a single token without receiving a new token value from an inbound packet, this effectively locks out other packets to enable the effect from the man-in-the-middle attack. The blue teams token value must reach an upper bound, whilst the red team tries to achieve a lower bound. When a node reaches either of the thresholds, it declares a state of win or defeat and terminates the decentralised program, posting the state to logs. Figure 9 gives a diagrammatic view of the scenario.

In this scenario example, with a starting value of 10, a walk across the network encompassing nodes {1,8,7,3,6,5,9,2,4} results to a token of value 12. However, as all nodes that start the simulation are able to send one packet before locking, multiple walks across the network can occur at a given time. Walks can be cut short by other walks, due to the node single packet lock, varying the simulation on each run, and at the point that each node reaches the upper or lower threshold, causing the eventual end of the simulation. It is worth noting that the scenario can be executed with a variety of underlying networking protocols and technologies, including both non-IP (e.g., NullNet) and IPv6 (e.g., RPL and 6LoWPAN) networking stacks. This allows the trainees to repeat simulations with varying numbers of nodes, using different network technologies, record results and compare different aspects of IoT networking. They gain a working understanding of a cyber security team structure and exposure to the high-level C programming of IoT devices.

### 6.1. Demonstration

**Figure 10:**
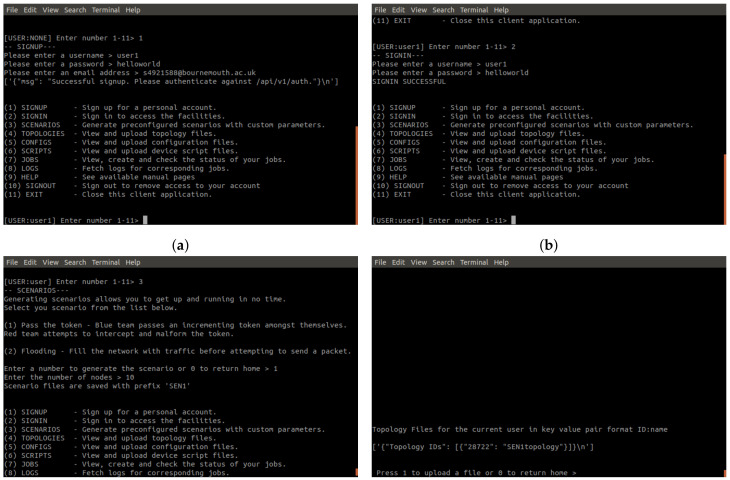

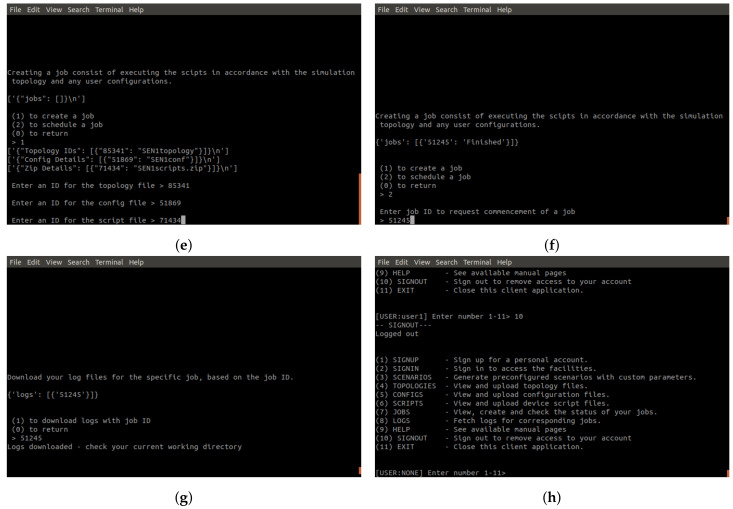
Python Wrapper Screenshots. (**a**) The signing up process. (**b**) The signing in process. (**c**) The creation of a scenario with parameters. (**d**) Topology page. You can see the scenario topology file already present. (**e**) Job creation. The user creates the job from the already uploaded files. (**f**) Job schedule. The user can enable the job to run, in doing so it’s state changes to finished. (**g**) Downloading the logs. A user can download all logs for each job. (**h**) The signing out process. You can see the username changes to NONE.

**Figure 11:**
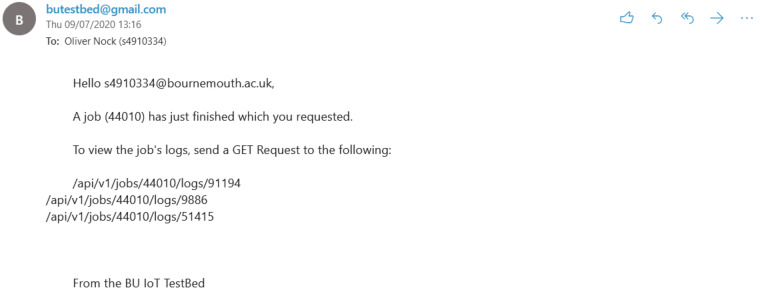
Example of communication with the user via email informing of log availability.

On first arrival at the system, users are required to make an account (Figure 10a). The username and password are then used for login and the email notification for job completion. Users are given the chance to generate the required files for a job, in the form of a scenario. These files are added to the users set of files so that custom variations of the scenario can be created. Such variations can include any parameters defined within the Cooja CSC file:- number of nodes, simulation duration time, assignment of particular nodes to scripts. Essentially the user is given the same level of control over the testbed as any adversary would hold over an actualized physical IoT Cyber Range. Alternatively, users can upload files, as seen in Figure 10d. Figure 10e,f show the creation and subsequent scheduling of the scenario job. In Figure 10e, the user picks from each file category to build a job, which is saved to the user account. Only when the user wishes to run the job do they utilise the job scheduling (Figure 10f). Once the job is completed, users will get an email informing them (Figure 11) and access to the logs is given in the client wrapper (Figure g).

### 6.2. Results

Utilising the demonstration, the testbed was operated under the following parameters:

- Network size: 10 Zolertia Motes
- Network protocol: Nullnet-an IPv6-less MAC networking protocol
- Mobility: Zero mobility across all nodes.
- All nodes commence execution at the same time.
- Simulation is terminated after 600 s (10 min).

The results Table 1, shows the metrics extracted from execution of the discussed scenario. Here 10 motes form 2 teams of varying size disparity. Sending packets via broadcast, every node records both the sent and received packets until the node reaches the defined threshold, where one team is declared the winner, or until the simulation times out.

These preliminary results serve to exhibit the utility of the testbed. In running the scenario discussed across several network sizes, it is shown that a novice user can generate quantifiable results. The flexibility of the testbed can be shown not only through the variable parameters, but also through the creative configuration by the user-to add a third team or increase the team disparity for example. The testbed encourages users to design future scenarios to address and combat the security challenges of IoT, closing the identified skills gap.

## 7. Conclusions and Future Work

As the technological paradigm of Internet of Things matures towards higher readiness levels, the gap in efficiently addressing corresponding cyber security aspects of IoT systems and the shortage in related IoT security skills become of increasing importance. This highlights the need for IoT experimenting and training facilities that are focused on security. In this work we presented an IoT Cyber Range; a user-focused IoT testbed designed to host multiple users and the execution of multiple training scenarios concurrently. We demonstrated its use via a red/blue team scenario involving a variant of man-in-the-middle attack using IoT devices.

On going work includes the extension of the IoT-CR towards its federation with the European network of cybersecurity centres of the H2020 ECHO project https://echonetwork.eu/, thus contributing to the build up of regional cybersecurity competence and capacity in Europe. The facility will be accompanied by readily available cyber-security training scenarios addressing nominal security issues of modern IoT networked systems. The efficiency of the scenarios in helping the trainees acquire new knowledge and skills will be evaluated by having diverse cohorts use the facility—ranging from low and moderate (e.g., undergraduate and postgraduate students) to high (e.g., IoT and cyber security professionals) expertise.

## Figures and Tables

**Table 1:** Results following a 10 mote Blue vs. Red simulation using the provided scenario.

Number of Nodes	Blue/Red Division	Avg. Packets Sent	Avg. Packets Received	Duration (Secs)	Winning Team
10	5/5	256,112	262,634	600	-
10	6/4	52,208	55,442	420	Blue
10	7/3	42,028	45,511	356	Blue
10	4/6	52,101	55,621	422	Red
10	3/7	41,986	42,420	321	Red
